# Application of direct peptide reactivity assay for assessing the  skin sensitization potential of essential oils

**DOI:** 10.1038/s41598-022-11171-2

**Published:** 2022-05-06

**Authors:** Elma Omeragic, Mirza Dedic, Alisa Elezovic, Ervina Becic, Belma Imamovic, Nebojsa Kladar, Haris Niksic

**Affiliations:** 1 University of Sarajevo-Faculty of Pharmacy, Zmaja od Bosne 8, 71 000 Sarajevo, Bosnia and Herzegovina; 2 University of Novi Sad-Faculty of Medicine, Hajduk Veljkova 3, 21000 Novi Sad, Serbia

**Keywords:** Plant sciences, Risk factors, Chemistry

## Abstract

Plant-derived products are frequently found as ingredients in cosmetics. However, the current data show non-neglectable skin sensitizing potential of these preparations suggesting an urgent need for data regarding their health safety profile. The aim of this study was to assess the skin sensitization potential of commercial essential oils by selected *Lamiaceae* species (*Lavandula angustifolia, Melissa officinalis, Mentha longifolia, Thymus vulgaris, Salvia officinalis,* and *Rosmarinus officinalis*) using a chemistry-based Direct Peptide Reactivity Assay (DPRA) in order to predict their potential allergic properties. In the DPRA assay, nucleophile-containing synthetic peptides (cysteine peptide and lysine peptide) were incubated with the test substance for 24 h. Depletion of the peptide in the reaction mixture was measured by high-pressure liquid chromatography (HPLC) using UV detection and the average peptide depletion data for cysteine and lysine was then calculated. *Menthae longifoliae aetheroleum* showed no or minimal reactivity with 4.48% cysteine depletion, *Rosmarini aetheroleum* and *Salviae aetheroleum* showed low reactivity with the 12.79% and 15.34% of cysteine depletion, respectively, while the other analyzed essential oils showed moderate reactivity with the cysteine depletion between 23.21 and 48.43%. According to DPRA predictive analysis, only *Menthae longifoliae aetheroleum* can be classified as negative, while all other essential oils may be classified as positive, thus having the potential to cause skin sensitization.

## Introduction

Contact dermatitis is described as a skin inflammation process occurring as a result of direct interaction of specific substance with the skin. Irritant contact dermatitis is more common and occurs when substances cause damage to skin immediately after exposure, without prior immunological sensitization. Allergic contact dermatitis occurs after prior exposure to an allergen and is a cell-mediated, type IV hypersensitivity reaction^1^. It is dose-dependent, but it also seems that the number of exposures, i.e. the accumulated dose, is important for the risk of contact allergy induction^2^.

Allergic contact dermatitis is very common condition in general public. The prevalence is estimated at 20.1% of the general population, being higher in adults (21.4%) than in children and adolescents (16.5%), and in women (27.9%) than in men (13.2%)^3^. After metals, the second largest cause of contact allergy is the exposure to cosmetic products, where fragrances and preservatives lead the list of allergens. Contact allergy to fragrances affects 1–3% of the general population^4^. Fragrances are not only found in cosmetic products, including care and decorative cosmetics and perfumes, but also in topical drugs, household products, foods and toys. Thus, it is quite difficult not to be exposed to these allergens in daily life.

The presence of 26 fragrance allergens in a cosmetic product has to be explicitly listed on the ingredients list. These substances and extracts are given in Annex III of the EU Cosmetic Regulation if present in sufficient concentrations to cause allergic response^2,5^. Thus, the individuals with known allergies to these ingredients can avoid them and their exposure is significantly decreased.

The study of contact dermatitis epidemiology of known allergens and the search for yet not specified allergens continues. The EU Commission Regulation 2017/1410 prohibited the use of 3- and 4-(4-hydroxy-4-methylpentyl) cyclohex-3-ene-1-carbaldehyde (HICC), as well as atranol and chloroatranol (natural components of oak tree moss (*Evernia prunastri*) and treemoss (*Evernia furfuracea*) in cosmetic products. These substances are the fragrance allergens responsible for the largest number of contact allergies cases in the past years. Cosmetic products containing them are prohibited on the EU market as of 23rd August 2021^6^. The Scientific Committee on Consumer Safety (SCCS) recommended to extend the current list of fragrance allergens on the labels of cosmetic products^2,7^.

Plant-based substances and extracts/essential oils are in the focus of the establishing of contact allergen potential. Several such extracts/essential oils, including some of the members of *Lamiaceae* family, have already been identified as established contact allergens, or not yet established due to the lack of data, but with a number of cases reported^1,2^.

Another very important point to consider is that European Directorate for the Quality of Medicines & HealthCare (EDQM) reported that in a study of cosmetic products on the European market, 7.7% of samples were non-compliant with legislative requirements due to a missing or false declaration of allergic fragrance compounds and 3.1% of samples sold as “perfume-free” contained fragrance compounds^8^.

The assessment of the potential of chemicals to cause skin sensitization typically involves the use of laboratory animals. The classical methods include tests on guinea pigs, such as the Guinea Pig Maximization Test (GPMT) of Magnusson and Kligman, the Buehler Test (OECD TG 406)^9^, or murine tests, such as the LLNA (OECD TG 429)^10^ and its three non-radioactive modifications—LLNA: DA (OECD TG 442A)^11^, LLNA: BrdU-ELISA, and BrdU-FCM (OECD TG 442B)^12^. The first two listed methods are based on assessing the induction and elicitation phases of skin sensitization. The murine tests, on the other hand, evaluate the induction response, and their additional advantage over the guinea pig tests is in terms of animal welfare together with an objective measurement of the induction phase of skin sensitization.

**Figure 1:**
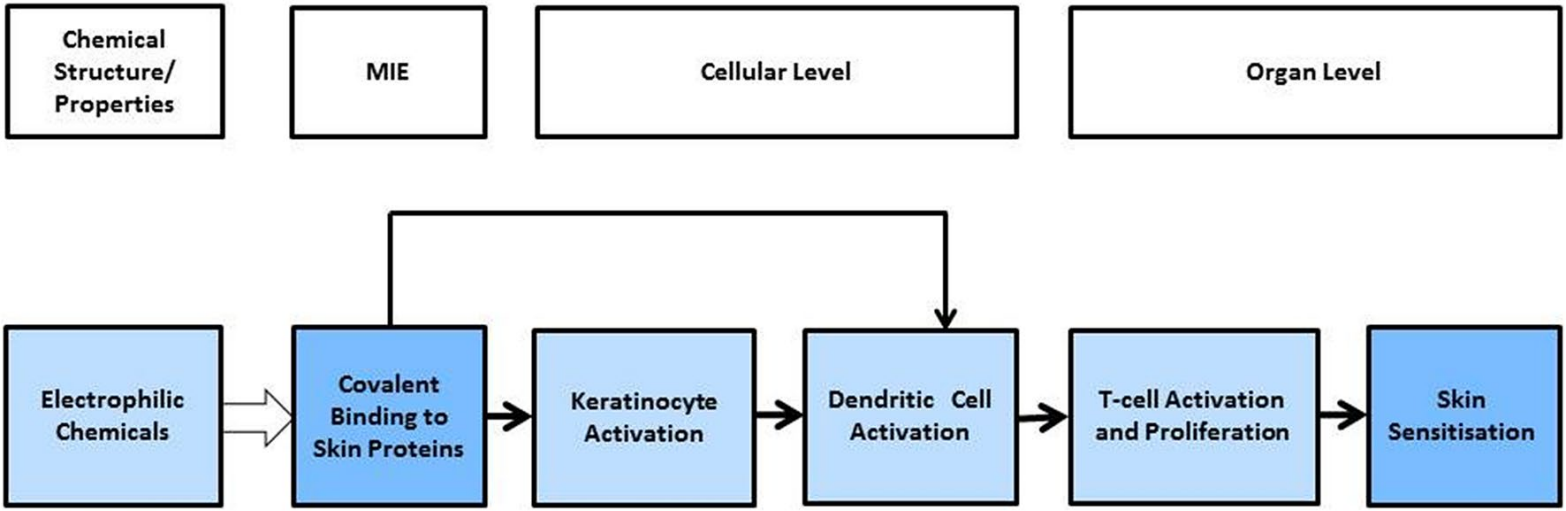
AOP: 40^48^.

As an alternative to animal tests, mechanistically-based *in chemico* and in vitro test methods (OECD TG 442C, 442D, 442E)^13,14^ which assess the first three key events (KEs) of the skin sensitization adverse outcome pathway (AOP) can be used in evaluating the skin sensitization hazard potential of chemicals. The skin sensitization AOP40 is focused on chemicals that react with amino acid residues (i.e. cysteine or lysine). In this instance, the molecular initiating event (MIE) is the covalent binding of electrophilic substances to nucleophilic centers in skin proteins. The second KE in this AOP takes place in the keratinocytes and includes inflammatory responses, as well as changes in gene expression associated with specific cell signaling pathways such as the antioxidant/electrophile response element (ARE)-dependent pathways. The third KE is the activation of dendritic cells, typically assessed by the expression of specific cell surface markers, chemokines and cytokines. The fourth KE is T-cell proliferation, and the adverse outcome is the presentation of allergic contact dermatitis (Fig. 1)^13^.

The primary aim of this study was to assess the skin sensitization potential of selected essential oils using *in chemico* Direct Peptide Reactivity Assay (DPRA) in order to predict their potential allergic properties. Secondly, we wanted to compare the DPRA results with the literature available results of skin sensitization potential of analyzed essential oils.

## Results

Table 1 summarises the individual components and chemical class distribution in selected essential oils.

**Table 1:** Chemical composition of selected essential oils.

Compounds	Percentage (%)							
		RI^a^	*Thymi aetheroleum*	*Rosmarini aetheroleum*	*Salivae aetherolum*	*Melissae aetheroleum*	*Lavandulae aetheroleum*	*Menthae aeteroleum*
Monoterpene hydrocarbons	α-pinene	939	1.54	11.18	7.59		0.18	5.89
	α-terpinene	1020	1.89	0.45	0.24			
	α-thujene	930	1.03	0.12	0.33		0.11	
	β-pinene	981	0.72	1.97	1.23			2.87
	Camphene	956	1.02	4.22	7.39			
	*cis*-β-ocimene	1050					3.30	
	γ-terpinene	1020	9.04	0.20	0.20		0.09	
	Limonene	1033	0.67	2.14	2.37		0.93	0.11
	Myrcene	990	1.53	1.51	0.98	0.15	0.91	0.48
	*p*-cymene	1029	29.93	3.58	1.57	0.53		
	Sabinene	974						0.68
	Terpinolene	1008						0.35
	*trans*-β-ocimene	1049					4.41	
	Other	–	0.33	0.60	1.20	0.30	0.81	
Monoterpene alcohols	α-terpinole	1196	0.37	5.55	0.34		2.80	
	Borneol	1176	1.73	4.99	3.11	2.13	1.95	0.52
	Carvacrol	1298	3.60	0.08	0.07			
	Citronellol	1228				1.48		
	Geraniol	1252				6.56		
	Lavandulol	1166					3.78	
	Linalool	1100	2.38	1.39	0.46		37.87	
	Nerol	1226				1.50		
	Terpinene-4-ol	1183	0.87	1.18			4.92	
	Thymol	1292	36.69		0.09	0.90		1.20
	Other	–	1.40	0.40	0.17	2.70	0.70	
Monoterpene oxides	1,8-cineole	1037	1.39	41.94	13.41	0.45	0.35	9.30
	α-thujone	1109			25.23	0.97		
	β-thujone	1121			7.42	0.27		
	Camphor	1152	0.65	16.31	19.27			
	*Cis*-linalool oxide	1038					1.56	
	Citronellal	1155				5.42		
	Geranial	1270				27.71		
	Neral	1241				19.54		
	Piperitenone	1343						2.43
	Piperitone oxide	1170						59.99
	*trans*-linalool oxide	9.21					1.16	
	Other	–		0.50	0.35		2.20	6.24
Other monoterpenes	Bornyl acetate	1286	0.30	0.53	1.86			
	Geranyl acetate	1378				4.14	1.44	
	Lavandulyl acetate	1284					8.31	
	Linalyl acetate	1251					12.14	
	Carvacrol methyl ether	1241	0.37					
	Methyl citronellate	1259				0.66		
	Thymol methyl ether	1231	0.32	0.32		3.70		
	Other	–			0.20	0.84	0.70	
Aliphatic compounds	1-octen-3-ol	981	0.72			0.88	0.56	
	3-octanol	997	0.12	0.08			0.33	1.27
	3-octanone	986	0.08					
	Methyl 2-methyl Butyrate	780	0.38					
	Other	–				2.70	2.46	
Sesquiterpenes	α-humulene	1633			0.80	0.35		
	β-caryophyllene	1420	0.44	0.33	0.12	3.77	1.08	4.20
	Germacrene D	1490						3.70
	Other	–				0.45	0.20	0.35
Sesquiterpenes alcohols		–	0.1		3.30	1.70	0.10	
Sesquiterpene oxides	Caryophyllene oxide	1582	0.39	0.13		10.20	2.50	0.34
	Other	–		0.3	0.40		0.20	0.08
Diterpenes					0.30			
Total identified			100.00	100.00	100.00	100.00	100.00	100.00

^a^Retention index calculated relative to elution time of C9-C24 n-alkane from HP 5MS column.

The chromatograms of the reaction mixtures of cysteine peptide and essential oils are shown in Table 2.

**Table 2:**
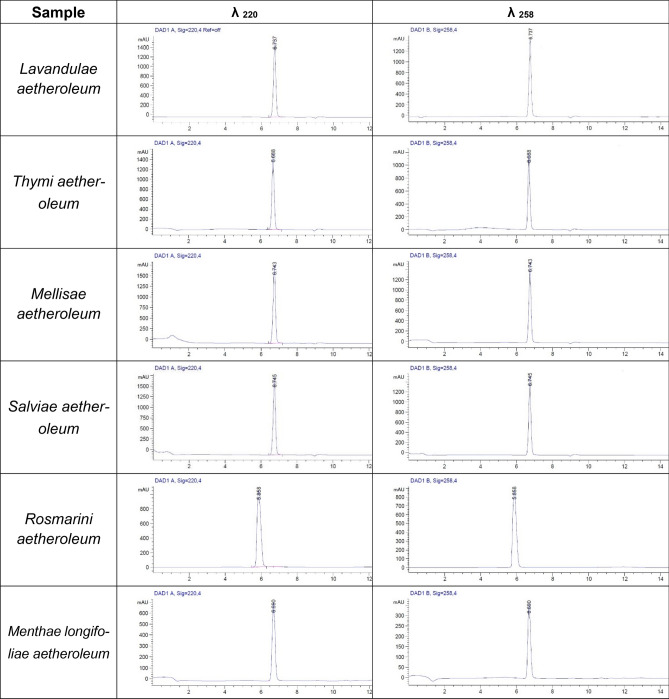
The chromatograms of the reaction mixtures of cysteine peptide and essential oils.

The proposed liquid chromatography based analytical approach for determination of skin sensitization potential of evaluated essential oils was found as an efficient method for the stated purpose. All acceptance criteria for the cysteine run were met and the results are shown in Table 3.

**Table 3:** Acceptance criteria**.**

Criteria	Accepted value	Found value
Calibration linearity (r^2^)	0.990	0.998
Mean peptide concentration of reference controls A	0.50 ± 0.05 mM	0.54 mM
The mean percent peptide depletion value of the three replicates for cinnamic aldehyde	60.80%-100%	82.32%
Standard deviation for percent cysteine depletion	< 14.9%	4.95
Stability of reference controls over analysis time	< 15.0%	6.41
Maximum standard deviation of sample replicates	< 14.9%	0.16–14.20
The mean of the peptide concentrations of the three appropriate reference controls C	0.50 ± 0.05 mM	0.54 mM
Peak purity indicator: area ratio 220/258	90% < mean area ratio of control samples < 110%	93.16 < mean area ratio of control samples < 108.6

As discussed in DPRA European Centre for the Validation of Alternative Methods (ECVAM) Validation Study Report in the DPRA test some chemicals, or the reaction products formed following incubation with the peptide, may interfere with either the cysteine peptide or the lysine peptide determination (a phenomenon referred to as co-elution) because they elute at the same time of the peptide^15^. According to the protocol^16^ co-elution of chemical and peptide may be explored by looking at the UV spectrum at 258 nm and calculating the area ratio of 220/258 (Peak purity indicator). Area ratio 220/258 for the lysine run showed that essential oils co-eluted with the lysine peptide. In the case of co-elution with the lysine peptide, the value for the lysine peptide depletion should be ignored^15,16^ and the Cysteine 1:10-only prediction model could only be used (Table 4).

**Table 4:** Cysteine 1:10-only prediction model**.**

Cysteine (Cys)% depletion	Reactivity class	DPRA prediction
0% < cysteine (Cys)% depletion < 13.89%	No or minimal reactivity	Negative
13.89% < cysteine (Cys)% depletion < 23.09%	Low reactivity	Positive
23.09% < cysteine (Cys)% depletion < 98.24%	Moderate reactivity	
98.24% < cysteine (Cys)% depletion < 100%	High reactivity	

The results of the conducted study have showed variable skin sensitization potential of the analyzed essential oils recorded in the DPRA. Specifically, the percentage of cysteine depletion in each sample replicates is shown in Table 5.

**Table 5:** Percent cysteine depletion in each sample replicates**.**

Sample	Peak area in replicate	RSD (%)	Percentage of cysteine depletion	DPRA prediction
*Lavandulae aetheroleum*	10,179.95	0.16	42.22	Positive
*Thymi aetheroleum*	12,987.75	2.24	26.28	Positive
*Melissae aetheroleum*	13,528.1	5.09	23.21	Positive
*Salviae aetheroleum*	14,915.05	14.20	15.34	Positive
*Rosmarini aetheroleum*	15,363.2	2.80	12.79	Positive
*Menthae longifoliae aetheroleum*	16,910.15	3.37	4.84	Negative

## Discussion

In this study, we used DPRA to predict human skin sensitization potential of commercial essential oils *Lamiaceae* species, namely *Lavandula angustifolia*, *Melissa officinalis*, *Mentha longifolia*, *Thymus vulgaris*, *Salvia officinalis* and *Rosmarinus officinalis* originating from Bosnia and Herzegovina (BiH). These species were selected for the analysis according to their wide geographical distribution and frequent use in formulation of various cosmetic and topical preparations and aromatherapy^17^.

The reactivity of the six essential oils toward the cysteine (peptide depletion ratio) is shown in Table 5.

The recorded percentage of cysteine depletions was increasing in the following order *Menthae longifoliae aetheroleum* (4.84%) < *Rosmarini aetheroleum* (12.79%) < *Salviae aetheroleum* (15.34%) < *Melissae aetheroleum* (23.21%) < *Thymi aetheroleum* (26.28%) < *Lavandulae aetheroleum* (42.22%).

*Menthae longifoliae aetheroleum* showed no or minimal reactivity, *Rosmarini aetheroleum* and *Salviae aetheroleum* showed low reactivity, while other essential oils showed moderate reactivity with cysteine peptide. For the moderate reactivity, between 23.21 and 48.43% depletion was observed for cysteine peptide. The essential oils classified as low reactive showed 12.79% (*Rosmarini aetheroleum*) and 15.34% (*Salviae aetheroleum*) depletion of cysteine peptide. According to DPRA predictive analysis, only *Menthae longifoliae aetheroleum* can be classified as negative, while all other essential oils may be classified as positive and have the potential to cause skin sensitization.

The results of DPRA test were expected bearing in mind the phytochemical status of each essential oil (Table 1). According to results of phytochemical composition the main compounds of *Rosmarini aetheroleum* are 1,8-cineole (41.94%), camphor (16.31%) and of *Salivae aetheroleum* thujone (25.23%) and 1,8-cineole (13.40%). The main compounds recorded in *Lavandulae aetheroleum* are linalool (37.87%) and linalyl acetate (12.14%), in *Melissae aetheroleum* geranial (27.71%), neral (19.52%), caryophyllene oxide (10.20%) and geraniol (6.56%), in *Thymi aetheroleum thymol* (36.63%) and p-cymene (29.93%) and in *Menthae longifoliae aetheroleum* piperitone oxide (59.99%) and 1,8-cineole (9.30%). Linalool^18,19^, citral^20^, p-cymene^21,22^, geraniol^23^ and thymol^24^ are known as substances with some concern to cause skin sensitization. However, linalool is reported negative for DPRA^25^. It should be noted that placed in the context of the AOP, the DPRA evaluates key event 1 − the protein/peptide reactivity of a substance so this result is expected. Still, linalool is clasified as human sentisazer according to LLNA test (reported EC3 values were 30% and 46%)^26^.

Additionaly, when we analyzed the chemical characteristics of the main compounds in tested essential oils we found that most of them including piperitone oxide and 1,8-cineole have an electrophilic property and would be able to react with cysteine but still the DPRA prediction for *Menthae longifoliae aetheroleum* was negative. Anyway, it is important to emphasise that the main aim of this study was to use DPRA test in characterising the skin sensitization effect of essential oils *in toto* and it is reasonable to expect that in such a complex sample, there could exist interaction (synergism and antagonism) among the chemicals in terms of their effect to cause skin sensitisation and the effect of a strong sensitizer (or extreme sensitizer) could be affected with some other chemical(s) so we didn't go into a theoretical analysis of so many unknown variables.

In addition, citral and geraniol are on the List A Perfume Allergens that includes fragrance chemicals, which according to existing knowledge, are most frequently reported and well-recognized consumer allergens. Moreover, linalool is on the List B Perfume Allergens that includes fragrance chemicals, which are less frequently reported and thus less documented as consumer allergens^27^. Our results of the phytochemical composition characterization of selected essential oils have shown that citral is one of the dominant compounds in *Melissae aetheroleum* (27.71%) and linalool in *Lavandulae aetheroleum* (37.87%), *Melissae aetheroleum* (2.13%), *Thymi aetheroleum* (2.38%), *Rosmarini aetheroleum* (1.39%), and *Salviae aetheroleum* (0.46%). Neither citral nor either linalool are identified in *Menthae aetheroleum* (Table 1). According to the European Medicine Agency (EMA) assessment report of *Rosmarini aetheroleum*, when applied topically, camphor as one of its main components can produce irritation of the skin, eyes, and mucous membranes of the respiratory tract^28^.

A recent literature review has shown that *Lavandulae aetheroleum* has been associated with allergic contact dermatitis of the hands in beauticians^29^, as well as with airborne contact dermatitis which is caused by *Lavandulae aetheroleum* used in aromatherapy^30^ and photoallergic contact dermatitis caused by topical drugs such as topical ketoprofen preparations^31^. In another study Warshaw et al.^32^ reviewed data collected by two large patch-test groups, one in North America and the other in Central Europe. They found that the prevalence of allergic reaction in the North American group was 2% for *Menthae piperitae* and *Lavandulae angustifoliae* essential oils while for *Menthae alternifoliae* essential oil the prevalence of allergic reaction was 5%.

Hagvall and Christensson^33^ also showed that oxidized *Lavanduae aetheroleum* causes the frequency of positive patch test reactions of 2.8% among the tested patients, while oxidized linalool and oxidized linalyl acetate showed 3.3% and 2.2% positive patch reaction among the tested patients, respectively. The result of this study was in accordance with the result of the Hagvall et al.^34^ study who found that the pure *Lavanduale aetheroleum* is a weak skin sensitizer, while the oxidized sample was classified as a moderate sensitizer in the local lymph node assay. Furthermore, patch testing showed positive reactions to air-exposed lavender oil and also to oxidized linalyl acetate in patients with contact allergy to oxidized linalool. In addition, studies showed that a significant amount of allergenic hydroperoxides are formed from air-exposed fragrance terpenes, i. e., limonene, linalool, and linalyl acetate. Hydroperoxides lead to form the secondary products of oxidation with various allergenic potentials, such as epoxides, alcohols, and carbonyl compounds which are strong reactive electrophiles^35^ and can contribute to the KE 826 (Keratinocytes Activation). We found a relatively high content of linalyl acetate (12.14%) in tested *Lavandulae aetheroleum* and it could contribute to the moderate reactivity of *Lavandulae aetheroleum* in DPRA test. Another study showed that geraniol has a different oxidation pattern compared to those of linalool and limonene. As a product of geraniol autooxidation, mainly hydrogen peroxide and the aldehydes geranial and neral are formed^33^. Geranial and neral have a strong electrophilic property because of their α, β-unsaturated carbonyl functionality^36^.

Amirghofran et al. study data showed the suppressive effects of thymol and carvacrol on dendritic cell maturation and function, as well as T cell responses^37^. Also, thymol doesn't have electrophilic property^38^ so it as one of the main compounds in *Thymi aetheroleum* probably couldn’t contribute to the first three KE of AOP which is on the basis of the DPRA test. The second major compound of *Thymi aetheroleum* is *p*-cymene that is in the literature characterized as a minor allergen in tea tree oil^39^. Para-cymene also had nucleophilic characteristics so the skin sensitization properties of analyzed *Thymi aeteroleum* may be attributed to some other ingredients of this essential oil or may be the consequence of their synergistic effects.

EMA also reported in the assessment of *Thymi aetheroleum* that in the literature, there is information on cases of skin irritation and allergic reactions, due to the content of thymol^40^.

It is also reported that *Rosmarini aetheroleum* can cause skin irritation and photosensitivity^41^ or that is recognized as an essential oil that has allergenic potential^23^. A few case studies also reported positive patch test results for the rosemary leaf extract^42,43^.

The EMA assessment report on *Salviae aetheroleum* recognizes this essential oil as a moderate skin irritant and does not recommend its usage in aromatherapy^44^.

Data on in vivo studies dealing with the contact allergic dermatitis caused either by *Melissae* or *Menthae longifloiae aetheroleum* were not available.

In the European Union (EU), cosmetic products are regulated by the European Commission (EC) Cosmetics Regulation no. 1223/2009, as amended, for which certain provisions relate specifically to the use of essential oils in cosmetic products. The Regulation requires that a cosmetic product has undergone a safety assessment based on relevant scientific information before being placed on the market. Checking the purity and composition of the essential oil, as well as the method of storage is necessary in assessing the safety profile. According to the requirements of the European Pharmacopoeia, essential oils require light protection in a well-closed and fully filled container. Photoisomerization, photocyclization, oxidation, peroxidation, and degradation of alcohols and ketones by hydrolysis are occurring due to unsuitable storage conditions. The safety of essential oils and individual compounds can be altered by decomposition. To avoid such consequences of decay, International Organization for Standardization (ISO) prescribes standards for essential oils. Standard ISO/TS 210: 2014 prescribes general rules for packaging, conditioning, and storage of essential oils^45^.

The safe use of essential oils in cosmetic products also depends on the quality of the raw materials and the extraction methods used to obtain the essential oil. It is often assumed that essential oils represent no hazard because they are of natural origin, obtained from plants. Only essential oils that meet pharmacopoeial quality standards could be used for medicinal purposes, unlike the use of essential oils in cosmetic products that are sometimes well below pharmacopoeial standards. Certain essential oils, depending on the dose, can cause skin reactions (essential oil of cinnamon, basil, mint, clove, niaouli, thyme, marjoram, etc.). Furthermore, essential oils obtained from lemon, bergamot, and bitter orange are phototoxic. Due to the complexity of these natural products, all ingredients that may act synergistically or antagonistically with each other should be evaluated from the toxicological and biochemical aspect^46^.

## Materials and methods

### Chemicals and reagents

Cinnamaldehyde (natural, ≥ 95%, FG), trifluoroacetic acid (purity 99%), ammonium acetate and ammonium hydroxide (28–30%) were purchased from Sigma-Aldrich Chemie GmbH, Germany. L-lysine (Purity: > 95%) and l-cysteine (Purity: > 95%) were purchased from JPT, Germany. Acetonitrile (for HPLC-SUPER GRADIENT Reag. Ph. Eur., ACS water < 30 ppm-suitable for UPLC/UHPLC instruments) was purchased from VWR Prolabo Chemicals Pennsylvania, United States of America. Sodium phosphate, monobasic monohydrate and sodium phosphate, dibasic heptahydrate were purchased from Kemika, Zagreb, Croatia. HPLC grade water was obtained using Arium Mini Sartorius (Goettingen, Germany) water purification systems.

### Essential oils

The essential oils used in this study, *Lavandulae angustifoliae aetheroleum*, *Melissa officinalis aetheroleum*, *Mentha longifoliae aetheroleum*, *Thymi vulgaris aetheroleum*, *Salviae officinalis aetheroleum* and *Rosmarini officinalis aetheroleum* originating from BiH, were purchased in year 2021 as commercial preparations of 100% purity from the private supplier. Geographical and botanical origin was claimed by the supplier. Samples were stored in glass bottle in the dark at 4 °C.

### Phytochemical characterization

Phytochemical analysis of the essential oils was carried out using a GC–MS 6890 N/5975B system (Agilent Technologies, Inc., Santa Clara, United States). Separations were performed on an HP 5MS column 30 m × 0.25 mm; film thickness 0.25 μm (Agilent). Helium was used as a carrier gas with the flow rate 1 mL min^−1^ and the temperature programs were 50 °C to 280 °C at a rate of 10 °C/min until 130 °C and 130–280 °C at a rate of 12 °C/min, respectively with split ratio, 1:10. The components of the essential oil were identified by obtained GC–MS spectra and retention indices (RI) relative to C8-C20 n-alkanes. For the calculation of the RI, the GC of the essential oil with C8-C24 *n*-alkane mixture were run and noted the retention times of alkane that was detected before and after an essential oil constituent. Then the Eq. (1) was used:

1 $$RI=100 \cdot n+100 \cdot \left[\left(\frac{log{t}_{x}-log{t}_{n}}{log{t}_{n+1}-log{t}_{n}}\right)\right]$$

_n_ and t_n+1_ are retention times of the reference *n*-alkane hydrocarbons eluting immediately before and after chemical compound “x”; t_x_ is the retention time of compound “x”.

For the components, mostly sesquiterpenes and aliphatic compounds, for which reference substances were not available, the identification was performed by matching their retention times and mass spectra with those obtained from the authentic samples and/or The National Institute of Standards and Technology, known as the National Bureau of Standards (NIST/NBS), Wiley libraries spectra as well as with literature data.

### Direct peptide reactivity assay

The DPRA is a chemistry-based assay. Nucleophile-containing synthetic peptides (cysteine peptide—Ac-RFAACAA-COOH; lysine peptide—Ac-RFAAKAA-COOH) are incubated with the test substance in the dark at 25 °C for 24 h. Depletion of the peptide in the reaction mixture is measured by high-pressure liquid chromatography (HPLC) using UV detection. The average peptide depletion data for cysteine and lysine are then calculated. All necessary reagents were prepared according to the procedures that are given in the DB-ALM Protocol N°154: Direct Peptide Reactivity Assay (DPRA) for Skin Sensitization Testing^47^. Cysteine stock solution was prepared by dissolving 12.5 mg of cysteine peptide in 25 mL of pH = 7.5 phosphate buffer to make a 0.667 mM solution. Lysine stock solution was prepared by dissolving 12.9 mg of lysine peptide in 25 mL of pH = 10.2 ammonium acetate buffer to make a 0.667 mM solution. Working calibration solutions were prepared by dilution of stock solutions with 20% (v/v) acetonitrile: buffer in order to obtain concentrations ranging from 1 to 0.0167 mM. Cinnamaldehyde (100 mM solution in acetonitrile) was used as the positive control for the assay and was included in every assay run.

By testing the solubility, it was found that each essential oil was soluble in acetonitrile. The chemical composition of essential oils was used to calculate the approximate molecular weight of each essential oil and prepared 100 mM solutions fresh, immediately before analysis. Samples are prepared in triplicate for both peptides. One sample is prepared without peptide, to verify whether the test chemical absorbs at 220 nm and has a similar retention time as a peptide, and may interfere with the data analysis (co-elution control).

Analysis was performed using HPLC 1260 Infinity II LC System (Agilent Technologies, Inc., Santa Clara, United States) equipped with a binary pump integrated with two-channel degasser, autosampler, column oven, and diode-array detector (DAD) controlled by OpenLAB CDS ChemStation Edition C.01.10. (https://www.agilent.com/chem/suplies) Separations were performed on a Zorbax SB-C18 5 µm column, 4.6 mm × 150 mm (Agilent). Gradient elution was carried out with acetonitrile and water, using linear gradient elution from 10 to 25% mobile phase B over 10 min, followed by a rapid increase to 90% to remove other materials. The flow rate was 0.2 mL min^−1^. The mobile phase was degassed prior to and during analysis. Before analysis, the entire system was equilibrated at 30 °C with 50% phase A (0.1% (v/v) trifluoroacetic acid in water) and 50% phase B (0.085% (v/v) trifluoroacetic acid in acetonitrile) for at least 2 h. The column was re-equilibrated under initial conditions for 7 min between injections. The compounds of interest were monitored at 220 nm and at 258 nm.

### Data analysis

The percent depletion of the peptide is determined in each sample by measuring the peak area and dividing that by the mean peak area of the reference control C (Eq. 2)

2 $$Percent \; Peptide \; Depletion=\frac{1-Peptide \; Peak \; Area \; in \; Replicate \; Injection}{Mean \; Peptide \; Peak \; Area \; in \; Reference \; Control \; C}*100$$

The acceptance criteria according to the ECVAM DB-ALM: Protocol were monitored. Before applying an appropriate prediction model, the experimental data were evaluated with care regarding the possibility of co-elution (Table 6).

**Table 6:** The different scenarios possible and recommended approach.

Mean depletion values	No co-elution	Co-elution with cysteine alone orcysteine and lysine	Co-elution with lysine only
Less than 6.38%	Minimal reactivity	Inconclusive	Apply cysteine-only prediction model
Between 6.38 and 22.62%	Low reactivity	≥ Low reactivity	Apply cysteine-only prediction model
Between 22.62 and 42.47%	Moderate reactivity	≥ Moderate reactivity	Apply cysteine-only prediction model
More than 42.47%	High reactivity	High reactivity	Apply cysteine-only prediction model

## Conclusions

We confirmed that the result of the direct peptide reactivity assay on skin sensitization potential is in accordance with the data available in literature regarding this effect of essential oils. However, the simplicity of this test and its feature of being an animal-free choice for toxicity testing highlights the advantage of its application for predicting the contact allergen potential of plant-based substances and extracts/essential oils. However, we noticed some discrepancies when considering the phytochemical properties of the analyzed essential oils and DPRA results, which further reinforces the need to use a combination of all methods that assess all three key events of the skin sensitization adverse outcome pathway in evaluating the skin sensitization hazard potential of chemicals.

## Data Availability

The datasets generated and/or analyzed during the current study are available from the corresponding author on reasonable request.

## References

[1] Oliver B, Krishnan S, Rengifo Pardo M, Ehrlich A (2015). Cosmeceutical contact dermatitis—cautions to herbals. Curr. Treat. Options Allergy.

[2] sccs_o_073.pdf.

[3] Prevalence of contact allergy in the general population: A systematic review and meta‐analysis—Alinaghi—2019—Contact Dermatitis—Wiley Online Library. 10.1111/cod.13119 30370565

[4] Prevalence of contact allergy in the general population: A systematic review and meta‐analysis—Alinaghi—2019—Contact Dermatitis—Wiley Online Library. 10.1111/cod.13119. 10.1111/cod.13119 30370565

[5] Regulation (EC) No 12232009 of the European Parli.pdf.

[6] Commission Regulation (EU) 2017 1410-of .pdf.

[7] ConPolicy, Directorate-General for Internal Market, I., ECORYS & VVA. *Impact assessment study on fragrance labelling on cosmetic products: final report*. (Publications Office of the European Union, 2020).

[8] EDQM reports presence of allergenic fragrances in cosmetics sold as “perfume-free”|EDQM—European Directorate for the Quality of Medicines. https://www.edqm.eu/en/news/edqm-reports-presence-allergenic-fragrances-cosmetics-sold-perfume-free.

[9] 2021—Test Guideline No. 406 Skin Sensitisation Guinea .pdf.

[10] Test No. 429: Skin Sensitisation: Local Lymph Node Assay|READ online. *oecd-ilibrary.org* https://read.oecd-ilibrary.org/environment/test-no-429-skin-sensitisation_9789264071100-en.

[11] European Commission. Directorate General for Health and Consumers.—2012—Toxicity and assessment of chemical mixtures..pdf.

[12] OECD. *Test No. 442B: Skin Sensitization: Local Lymph Node Assay: BrdU-ELISA or –FCM*. (Organisation for Economic Co-operation and Development, 2018).

[13] Test No. 442E: In Vitro Skin Sensitisation: In Vitro Skin Sensitisation assays addressing the Key Event on activation of dendritic cells on the Adverse Outcome Pathway for Skin Sensitisation | READ online. *oecd-ilibrary.org* https://read.oecd-ilibrary.org/environment/test-no-442e-in-vitro-skin-sensitisation_9789264264359-en.

[14] OECD. *Test No. 442C: In Chemico Skin Sensitisation: Assays addressing the Adverse Outcome Pathway key event on covalent binding to proteins*. (Organisation for Economic Co-operation and Development, 2021).

[15] DPRA Validation Study Report.pdf.

[16] 154_P_ Direct Peptide Reactivity Assay (1).pdf.

[17] Herman A, Herman AP (2015). Essential oils and their constituents as skin penetration enhancer for transdermal drug delivery: A review. J. Pharm. Pharmacol..

[18] Audrain H (2014). Allergy to oxidized limonene and linalool is frequent in the UK. Br. J. Dermatol..

[19] Contact sensitization to hydroperoxides of limonene and linalool: Results of consecutive patch testing and clinical relevance—Dittmar—2019—Contact Dermatitis—Wiley Online Library. 10.1111/cod.13137 PMC6587870 30378131

[20] Hagvall L (2020). Contact allergy to citral and its constituents geranial and neral, coupled with reactions to the prehapten and prohapten geraniol. Contact Dermatitis.

[21] Giménez-Arnau E (2019). Chemical compounds responsible for skin allergy to complex mixtures: How to identify them?. Cosmet. Toiletries.

[22] de Groot AC, Schmidt E (2016). Tea tree oil: contact allergy and chemical composition. Contact Dermatitis.

[23] Sindle A, Martin K (2021). Art of prevention: Essential oils—natural products not necessarily safe. Int. J. Women’s Dermatol..

[24] THYMUS VULGARIS (COMMON THYME) OIL|Substance. http://www.ewg.org/guides/substances/6029-THYMUSVULGARISCOMMONTHYMEOIL.

[25] Natsch A (2013). A dataset on 145 chemicals tested in alternative assays for skin sensitization undergoing prevalidation: 145 chemicals tested in alternative assays for skin sensitization. J. Appl. Toxicol..

[26] Kolle SN, Natsch A, Gerberick GF, Landsiedel R (2019). A review of substances found positive in 1 of 3 in vitro tests for skin sensitization. Regul. Toxicol. Pharmacol..

[27] 1. Introduction - European Commission. https://ec.europa.eu/health/scientific_committees/opinions_layman/perfume-allergies/en/l-3/1-introduction.htm.

[28] Assessment report on Rosmarinus officinalis L ... / assessment-report-on-rosmarinus-officinalis-l.pdf / PDF4PRO. *PDF4PRO* https://pdf4pro.com/amp/view/assessment-report-on-rosmarinus-officinalis-l-a3669.html (2018).

[29] De Mozzi P, Johnston GA (2014). An outbreak of allergic contact dermatitis caused by citral in beauticians working in a health spa. Contact Dermatitis.

[30] Schaller M, Korting HC (1995). Allergic airborne contact dermatitis from essential oils used in aromatherapy. Clin. Exp. Dermatol..

[31] Goiriz R, Delgado-Jiménez Y, Sánchez-Pérez J, García-Diez A (2007). Photoallergic contact dermatitis from lavender oil in topical ketoprofen. Contact Dermatitis.

[32] Warshaw EM (2017). Positive patch-test reactions to essential oils in consecutive patients from North America and Central Europe. Dermatitis.

[33] Hagvall L (2007). Fragrance compound geraniol forms contact allergens on air exposure. Identification and quantification of oxidation products and effect on skin sensitization. Chem. Res. Toxicol..

[34] Hagvall L, Sköld M, Bråred-Christensson J, Börje A, Karlberg A-T (2008). Lavender oil lacks natural protection against autoxidation, forming strong contact allergens on air exposure. Contact Dermatitis.

[35] Rudbäck J, Bergström MA, Börje A, Nilsson U, Karlberg A-T (2012). α-Terpinene, an antioxidant in tea tree oil, autoxidizes rapidly to skin allergens on air exposure. Chem. Res. Toxicol..

[36] White B, Evison A, Dombi E, Townley HE (2017). Improved delivery of the anticancer agent citral using BSA nanoparticles and polymeric wafers. NSA.

[37] Amirghofran Z (2016). In vitro inhibitory effects of thymol and carvacrol on dendritic cell activation and function. Pharm. Biol..

[38] Hamidi S (2019). A theoretical study of regio and stereoselectivity nitration of thymol and carvacrol using DFT approach. Moroc. J. Chem..

[39] de Groot, A. C. p-Cymene. In *Monographs in Contact Allergy* (CRC Press, 2019).

[40] Assessment report on *Thymus vulgaris* L., *Thymus zygis* L., aetheroleum. 29.

[41] EDQM reports presence of allergenic fragrances in cosmetics sold as “perfume-free”|EDQM—European Directorate for the Quality of Medicines.

[42] Inui S, Katayama I (2005). Allergic contact dermatitis induced by rosemary leaf extract in a cleansing gel. J. Dermatol..

[43] González-Mahave I, Lobesa T, Del Pozo MD, Blasco A, Venturini M (2006). Rosemary contact dermatitis and cross-reactivity with other labiate plants. Contact Dermatitis.

[44] Assessment report on *Salvia officinalis* L., folium.pdf.

[45] Sarkic A, Stappen I (2018). Essential oils and their single compounds in cosmetics—A critical review. Cosmetics.

[46] Abelan, U. S. *et al.* Potential use of essential oils in cosmetic and dermatological hair products: A review. *J. Cosmet. Dermatol.* n/a. 10.1111/jocd.14286 34129742

[47] DPRA OECD.pdf.

[48] eeeSkin_sensitisation_graph_neu_2.jpg (960×720). https://aopwiki.org/system/dragonfly/production/2016/11/29/eeeSkin_sensitisation_graph_neu_2.jpg.

